# Exploration of an adaptive proton therapy strategy using CBCT with the concept of digital twins

**DOI:** 10.1088/1361-6560/ada684

**Published:** 2025-01-17

**Authors:** Chih-Wei Chang, Zhen Tian, Richard L J Qiu, H Scott Mcginnis, Duncan Bohannon, Pretesh Patel, Yinan Wang, David S Yu, Sagar A Patel, Jun Zhou, Xiaofeng Yang

**Affiliations:** 1 Department of Radiation Oncology and Winship Cancer Institute, Emory University, Atlanta, GA 30308, United States of America; 2 Department of Radiation and Cellular Oncology, University of Chicago, Chicago, IL 60637, United States of America

**Keywords:** Digital twins, adaptive proton therapy, CBCT

## Abstract

*Objective.* This study aims to develop a digital twin (DT) framework to achieve adaptive proton prostate stereotactic body radiation therapy (SBRT) with fast treatment plan selection and patient-specific clinical target volume (CTV) setup uncertainty. Prostate SBRT has emerged as a leading option for external beam radiotherapy due to its effectiveness and reduced treatment duration. However, interfractional anatomy variations can impact treatment outcomes. This study seeks to address these uncertainties using DT concept to improve treatment quality. *Approach*. A retrospective study on two-fraction prostate proton SBRT was conducted, involving a cohort of 10 randomly selected patient cases from an institutional database (*n* = 43). DT-based treatment plans were developed using patient-specific CTV setup uncertainty, determined through machine learning predictions. Plans were optimized using pre-treatment CT and corrected cone-beam CT (cCBCT). The cCBCT was corrected for CT numbers and artifacts, and plan evaluation was performed using cCBCT to account for actual patient anatomy. The ProKnow scoring system was adapted to determine the optimal treatment plans. *Main Results.* Average CTV D98 values for original clinical and DT-based plans across 10 patients were 99.0% and 98.8%, with hot spots measuring 106.0% and 105.1%. Regarding bladder, clinical plans yielded average bladder neck V100 values of 29.6% and bladder V20.8 Gy values of 12.0cc, whereas DT-based plans showed better sparing of bladder neck with values of 14.0% and 9.5cc. Clinical and DT-based plans resulted in comparable rectum dose statistics due to SpaceOAR. Compared to clinical plans, the proposed DT-based plans improved dosimetry quality, improving plan scores ranging from 2.0 to 15.5. *Significance.* Our study presented a pioneering approach that leverages DT technology to enhance adaptive proton SBRT, potentially revolutionizing prostate radiotherapy to offer personalized treatment solutions using fast adaptive treatment plan selections and patient-specific setup uncertainty. This research contributes to the ongoing efforts to achieve personalized prostate radiotherapy.

## 1. Introduction

Ultra-hypofractionated radiotherapy, involving the delivery of radiation doses greater than 5 Gy per fraction, has emerged as the standard of care for prostate cancer external beam radiation therapy (RT) with low rates of side-effects (C *et al* 2022, Ma *et al* 2024). For instance, two recent clinical trials, 2STAR (NCT02031328) (Alayed *et al* 2019) and 2SMART (NCT03588819) (Ong *et al* 2023a), provides the guidelines for two-fraction prostate stereotactic body RT (SBRT). The ultra-hypofractionated approach offers significant advantages over conventional RT regimens which typically involve smaller daily fractions administered over a more extended treatment course. Ultra-hypofractionation reduces the overall treatment time, providing greater convenience and cost-effectiveness for patients, and exploits the radiobiological principles of hypofractionation, potentially enhancing the therapeutic ratio (Hannan *et al* 2016, Kishan *et al* 2019). The advent of proton pencil beam SBRT has further revolutionized the delivery of ultra-hypofractionated RT for prostate cancer (Bryant *et al* 2016). This cutting-edge technique combines robust treatment planning (Liu *et al* 2012) with advanced image-guided systems (Zwart *et al* 2022), enabling the precise administration of ultra-high radiation doses within five fractions. By leveraging the unique physical properties of protons, proton SBRT can potentially offer the unparalleled capability for sparing healthy tissues compared to conventional photon-based modalities.

As emerging clinical data continue to validate the effectiveness and safety of prostate SBRT management, efforts are underway to further optimize treatment quality and outcomes (Tilbæk *et al* 2023, Shen *et al* 2024). Two critical factors that can significantly enhance the therapeutic potential of prostate SBRT (Mancosu *et al* 2016) are (1) mitigating geometrical uncertainties and (2) maximizing dose conformity to targets while sparing the surrounding organs at risk (OARs). Geometrical uncertainties, arising from variations in anatomy between treatment fractions and positional inaccuracies, can compromise target coverage and increase the risk of toxicity. Adaptive radiotherapy strategies (Bobić *et al* 2021, Paganetti *et al* 2021), which involve modifying the treatment plan based on daily imaging, offer a promising solution for these uncertainties. By continuously adapting the plan to the patient’s anatomy, target coverage can be maintained while minimizing unnecessary doses for healthy tissues. However, the current treatment planning techniques are limited by computational constraints, making real-time adaptive proton therapy challenging (Chang *et al* 2024). This work aims to investigate an image-guided digital twin (DT) framework to integrate patient-specific clinical target volume (CTV) positional setups with uncertainty and proton robust plan optimization. The goal is to create multiple treatment plans, enabling online decision-making for selecting the optimal treatment plan on the actual treatment day.

A DT concept framework (Kapteyn *et al* 2021) leverages multiphysics models to forecast event progression and employs data assimilation techniques to accurately evaluate system states, thereby delivering recommendations for appropriate control actions. This framework has been explored as an *in-silico* patient modeling for personalized medicine (Björnsson *et al* 2019, Hormuth *et al* 2021). Nonetheless, using DT for diagnostic and therapeutic decisions necessitate consideration of multiple factors (Wu *et al* 2022), and DT for health is still in its infancy (Katsoulakis *et al* 2024). In this study, we investigate the potential of using the concept of DTs for potential online proton adaptive therapy using all available patient images and patient-specific CTV.

The proposed DT framework seeks to enhance the efficiency and accuracy for adaptive proton therapy techniques by leveraging advanced computational methods and patient-specific CTV setup uncertainty. This patient-specific uncertainty allows different patients to have different CTV margins during proton robust optimization, while the conventional proton planning uses a fixed margin for robust optimization (Unkelbach *et al* 2018, Biston *et al* 2020). By using the patient-specific CTV setup uncertainty and generating robust proton treatment plans tailored to potential anatomical variations, this approach aims to facilitate plan adaptation based on the patient’s daily imaging data. The generated treatment plans are based on the conventional treatment plans but with different CTV robust optimization margins. Through the creation of multiple treatment plans, the framework empowers clinicians to potentially make informed decisions regarding the most appropriate plan for the patient’s anatomy on the day of treatment. This approach can potentially enhance target coverage, minimize dose to OARs, and streamline the adaptive radiotherapy workflow, potentially reducing treatment times and improving overall efficiency, compared to the conventional online proton adaptive therapy (Chang *et al* 2024). According to the authors’ best knowledge, this research pioneers the application of DT in external beam radiotherapy. The primary contributions of this study can be categorized into two distinct dimensions, both of which are geared toward assessing the feasibility of clinical implementation:

• The proposed framework demonstrates how to leverage the concept of DTs to facilitate online proton adaptive therapy without requiring new multicriteria optimization. The framework create multiple prostate SBRT plans with patient specific CTV positional uncertainties using all available and adequately evaluated images including computed tomography (CT) and daily cone-beam CT (CBCT). By enabling the online treatment plan selection of the most suitable treatment plan from pre-approved candidates, the framework can potentially enhance healthy tissue sparing. This method streamlines the process by eliminating the need for online plan re-optimization, relying solely on online evaluation of existing plans.
• The proposed framework demonstrates how to assimilate CT and CBCT data from prior treatments to predict the most likely patient-specific CTV positional setups with uncertainty. This uncertainty leads to the creation of multiple patient-specific margin sets for proton robust optimization, resulting in several treatment plans and facilitating online selection of the most optimal plan for treatment.

## 2. Materials and methods

### 2.1. Treatment planning and two-fraction prostate SBRT

RayStation 2023B (RaySearch Lab., Stockholm, Sweden), was used to provide fast robust proton treatment planning and plan evaluation based on the same-day CBCT to enable real-time decision-making for delivering the optimal treatment plan. The treatment planning system (TPS) is deployed on a clinical GPU server with a single NVIDIA Quadro RTX 8000 and dual Intel® Xeon® Gold 6136 CPU. RayStation 2023B supports GPU-based deformable image registration for corrected CBCT generation that can be used for proton Monte Carlo (MC) dose calculation. The corrected cone-beam CT (cCBCT) processing workflow in RayStation 2023B (Thing *et al* 2022) employs a two-phase iterative approach. Initially, the system performs deformable registration between the CBCT and planning CT images, automatically creating a body contour on the CBCT that guides the registration process. This registration enables the creation of a two-dimensional histogram, from which peak values are detected to establish a piecewise linear mapping from CBCT values into the Hounsfield Unit scale. Subsequently, the process generates an artifact correction map by analyzing both the HU-converted CBCT and a deformed version of the planning CT. The system repeats these phases iteratively until the correction map reaches convergence. The cCBCT can be used for online proton treatment evaluation in 2 min (Chang *et al* 2023b). The treatment evaluation is achieved by calculating dose distribution on cCBCT, which represented patient anatomy on the treatment day and corrected CT numbers based on deformable image registration from planning CT. This method has been calibrated and demonstrated using quality assurance CT (QACT) with the evaluation metrics including proton water equivalent thicknesses and dosimetric gamma comparisons in our previous investigation (Chang *et al* 2023b).

**Figure 1.**
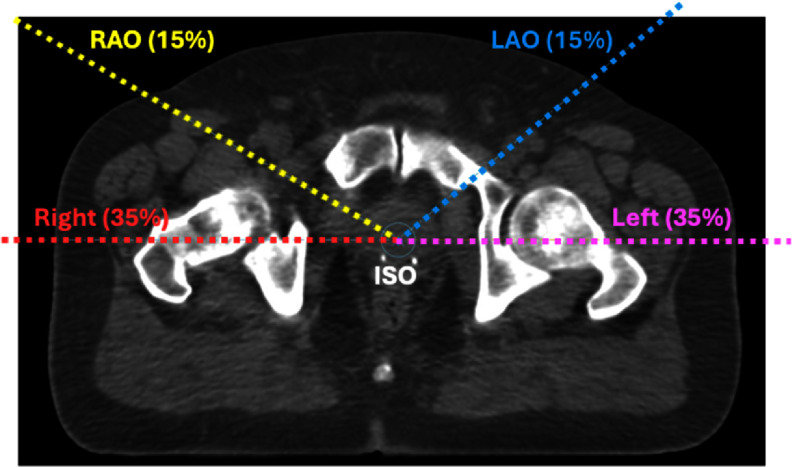
Beam arrangement of the two-fraction prostate SBRT using LAO, RAO, and bi-lateral beams (left and right) with the beam weighting of 15%, 15%, 35%, and 35%, respectively. The LAO and RAO beams uses gantry angles of 50° and 300°.

At our institution, prostate SBRT plans were optimized using four proton beams, including bi-lateral, left anterior oblique (LAO), and right anterior oblique (RAO) as shown in figure 1. The use of anterior-oblique beams can potentially limit the rectum doses (Moteabbed *et al* 2017). The beam model embedded a constant relative biological effectiveness (RBE) of 1.1, recommended by IAEA/ICRU (ICRU78 2007, IAEA 2008). Robust optimization was used for all clinical plans with 5 mm positional uncertainty (except 3 mm for posterior) and ±3.5% range uncertainty, resulting in 21 scenarios in each plan optimization.

To the best knowledge of the authors, there is yet to have proton-specific clinical trials regarding two-fraction prostate SBRT with published results; instead, we refer the planning dose constraints from the photon two-fraction prostate SBRT trials and use the proton RBE of 1.1. This work utilizes the OAR dosimetry constraints and prescription doses from the two-fraction prostate SBRT clinical trials, 2STAR (NCT02031328) (Alayed *et al* 2019) and 2SMART (NCT03588819) (Ong *et al* 2023a), to explore the feasibility of applying the proposed DT framework for adaptive proton therapy planning. The treatment delivery was 1 week apart for these two clinical trials. For the 2STATR trial, the prescribed dose was 26 Gy delivered in two fractions to the CTV, with a one-week break between fractions. The 2SMART trial included an additional dose-escalated boost up to 32 Gy to the gross tumor volume (GTV). Table 1 shows the dose constraints for OARs, including the bladder and rectum. Slightly higher dose limits were allowed for the bladder and rectum in the 2SMART trial to accommodate the GTV boost (Ong *et al* 2023b). In this work, we planned the prostate treatment with a simultaneously integrated boost (SIB) based on the prescription and clinical paraments from 2SMART in table 1. The 2STAR prescription and clinical goals were used for the prostate treatment without a SIB.

**Table 1.** Clinical parameters and dose constraints for two-fraction prostate SBRT treatment planning.

Clinical parameters	2STAR	2SMART
Prescription	26 Gy	32 Gy
Fraction (Fx)	2	2
Beams	4	4
CTV	D98 ⩾ 100%	D98 ⩾ 100%
	D0.03cc < 110%	D0.03cc < 110%
Bladder	V14.6 Gy < 15 cc	V14.6 Gy < 25 cc
	V20.8 Gy < 5 cc	V20.8 Gy < 10 cc
Urethra	N/A	D0.03cc < 33.8 Gy
		D10% < 30.4 Gy
Rectum	V13Gy < 7 cc	V13Gy < 7 cc
	V17.6 Gy < 4 cc	V17.6 Gy < 4 cc
	V20.8 Gy < 1 cc	V22Gy < 1 cc

Additionally, we contoured the bladder neck in this study, as it has been identified as strongly correlated with bladder toxicity (Hathout *et al* 2014). While there are no established dose constraints for the bladder neck, we utilized the dose statistics for this structure as a plan quality factor to determine the optimal treatment plan. All the CTV and OARs contours on CT and CBCT were created and reviewed by radiation oncologists to ensure the accuracy and precision for dosimetry analyses. All the CBCT images, used for treatment planning and plan evaluation, were corrected to match the CT numbers of planning CT acquired from the institutional Siemens scanner (Chang *et al* 2023b).

### 2.2. Patient imaging data

We utilized institutional CBCT image data to investigate treatment planning and delivery using the proposed DT framework for adaptive proton therapy. The CBCT images served three purposes: predicting patient-specific setup uncertainty for CTV, robust proton treatment planning, and treatment evaluation. Since our institutional was yet to start two-fraction prostate proton SBRT, the CBCT image sets were retrieved from 43 prostate cancer patients who received five-fraction SBRT with SpaceOAR hydrogel. Then we computed the center of mass (COM) coordinates for the CTV contours on each CBCT and selected two CBCT image sets with the largest COM distance to emulate the two-fraction SBRT treatment. This select ensured the maximum difference between the two CBCT image sets.

Six of those patients received a SIB for a dominant intraprostatic lesion (DIL). Each of the 43 patients had CBCT scans acquired on the Varian ProBeam® on-board imaging system, resulting in a total of 215 CBCT image sets. CBCT images were acquired using the tube voltage of 125 kVp and current of 176 mA, and images were reconstructed using Ram-Lak convolution kernel with resolution of 1.02 × 1.02 × 1.99 mm^3^ and 104 slices. We randomly selected 10 patients, including 2 patients with DIL SIB, from the institutional prostate SBRT database to demonstrate the proposed DT framework. The remaining patients were employed to train a machine learning model for predicting patient-specific CTV setup uncertainty. Additionally, we included CBCT images from another group of 49 prostate cancer patients who underwent conventional 28-fraction proton therapy to expand the image database and provide prior knowledge for inferring the underlying correlation between CBCT images and the CTVs, which were manually contoured by radiation oncologists on CBCT. Each of these 49 patients had daily CBCT imaging during treatment, contributing 1372 CBCT image sets. All patients had pre-treatment CT simulation images obtained from a Siemens SOMATOM Definition Edge scanner for initial treatment planning.

### 2.3. Adaptive proton treatment framework using the concept of DTs

**Figure 2.**
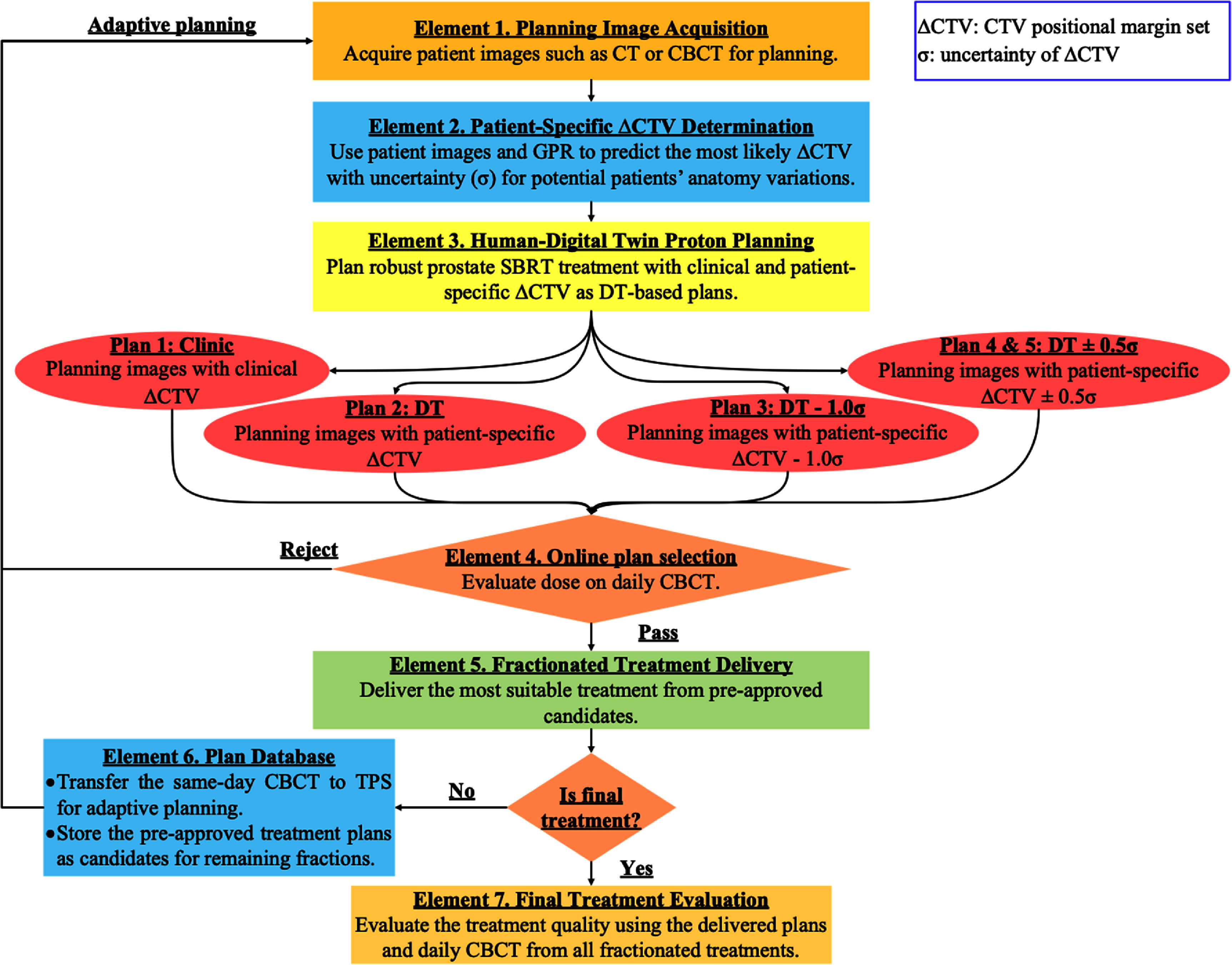
Overview of the proposed framework using the concept of digital twin (DT) for facilitating adaptive proton prostate SBRT treatment. The framework generates multiple treatment plans that enable the plan selection on the treatment day. Initially, patient images can be pre-treatment CT or CBCT for planning (Element 1). The Element 2 uses machine learning methods such as GPR to predict patient-specific CTV positional uncertainty margins, which will be used in Element 3 for proton robust planning. The clinical robust proton plan is obtained with a standard CTV setup uncertainty (Plan 1), and DT-based plans (Plan 2–5) are robustly optimized based on different patient-specific CTV setups with uncertainty. The most suitable treatment plan is determined by online plan evaluation using daily CBCT acquired before each treatment fraction delivery (Element 4–5). After the treatment delivery, daily CBCT will be sent to TPS for creating new plans for the consecutive treatment, and the existed approved plans are stored in the database as potential candidates for the remaining treatment (Element 6). Ultimately, the overall treatment quality will be evaluated (Element 7) using the delivered plans and daily CBCT images.

While the concept of DT in healthcare is still in its infancy, Katsoulakis *et al* (2024) proposed a definition that synthesizes various perspectives and descriptions found in the literature. Their definition includes that DT for healthcare can facilitate dynamic simulation of treatment strategies and monitors and predicts health outcomes. Building on their definitions, we propose a framework, illustrated in figure 2, that leverages DT concepts to facilitate adaptive proton therapy by utilizing patient imaging such as pre-treatment CT or daily CBCT (Element 1). To incorporate dynamic treatment strategies, a machine learning model, Gaussian process regression (GPR) (Ebden 2015), is implemented in Element 2 to predict patient-specific CTV positional uncertainties based on imaging data. These predictions (*σ*) are then used to derive multiple CTV positional margin sets for robust proton optimization. A CTV margin set includes the values for superior-inferior, left-right, and anterior–posterior positions. This process generates several DT-based treatment plans in Element 3 (Plans 2–5), each designed with varying patient-specific CTV margins tailored to the imaging data, along with a baseline plan (Plan 1) created using constant CTV margins in accordance with institutional clinical guidelines. Each plan optimization takes approximately 45 min using our institutional planning infrastructure and adheres to the planning goals outlined in table 1.

Element 4 demonstrates that online plan selection can be achieved by evaluating pre-approved plans (Plans 1–5) against daily CBCT to identify the most suitable option (Element 5), ensuring continuity without interrupting treatment. For treatment fractions beyond the first, Element 4 also incorporates an evaluation of prior treatment plans in conjunction with the current CBCT. Since the online evaluation process requires less than 2 min, the patient remains on the treatment table, avoiding delays associated with adaptive plan optimization. This approach allows for the delivery of treatment plans with smaller, patient-specific CTV setup margins compared to conventional constant margins. Following each fractionated treatment, same-day CBCT is sent to TPS for adaptive planning for subsequent treatment fractions, and approved plans are stored in the database for remaining scheduled treatment (Element 6). At our institution, it generally takes physicians approximate 10 min to contour CTV on CBCT. The new CBCT-based plans can be created since the 2STAR and 2MART clinical trials are 1 week apart between the treatment fractions. Finally, the treatment quality is assessed by evaluating delivered treatment plans and daily CBCT data based on the treatment history (Element 7). This framework aims to improve treatment precision and efficiency while maintaining high-quality care.

### 2.4. Gaussian process regression for predicting patient-specific CTV robust setups with uncertainty

All the CTV structures on CT and CBCT were manually contoured by radiation oncologists, and the femoral head structures were contoured by dosimetrists. Then we defined the reference positions for each contour that were used to quantify the image features. We first calculated COM coordinates as reference positions of each CTV. Subsequently, we constructed a bounding box encompassing the femoral heads, and delineated the reference femoral coordinates based on the vertices of this bounding box. The coordinate for the left femoral head (FemL) was defined as the vertex posterior and inferior to the patient’s midline, while the coordinate for the right femoral head (FemR) was defined as the vertex posterior and superior to the patient’s midline.

After defining all reference positions for CTV and femoral head contours, we defined the relative CTV position by using CTV coordinate subtracting FemL coordinate in left-right, anterior–posterior, and superior-inferior directions. The relative CTV position was then determined by subtracting the CTV coordinate from the FemL coordinate. Table 2 describes all the image features utilized to correlate with the relative CTV position. An in-house RayStation 2023B script was developed to calculate all features from images.

**Table 2.** Definition of image features utilized for constructing a GPR model that correlates with patient-specific relative CTV positions.

Image feature (GPR model parameter)	Description
1	Euclidean distance between FemL and FemR
2	Euclidean distance between CTV and FemL
3	Euclidean distance between CTV and FemR
4	Angle between the two vectors by CTV-FemL and CTV-FemR
5	Distance (Δ*X*) between FemR and CTV in left-right direction
6	Distance (Δ*Y*) between FemR and CTV in anterior–posterior direction
7	Distance (Δ*Z*) between FemR and CTV in superior-inferior direction

Our goal is to use image features to predict the relative CTV positions that can be used to determine the patient-specific CTV setup margins for the proton robust optimization. We utilized a MATLAB-based GPR package (Rasmussen and Nickisch 2010) to find the underlying correlation between the relative CTV position and image features extracted from both CT and CBCT scans. We are interested in a GPR model because it not only predicts mean value but also gives the uncertainty estimates. Each image feature in table 2 served as a GPR model parameter for predicting the relative position of CTV based on the input image. Ultimately, we obtained a GPR model to predict CTV positions for the first and second treatment fractions (Fx1 and Fx2), and we used them to determine the patient-specific CTV setup uncertainty margins. To prevent GPR model from predicting physically unreasonable CTV robustness margins, we imposed a lower bound of 1.5 mm due to inherent uncertainties arising from the coincidence of radiation and mechanical isocenters. The upper bound was set to 5.0 mm based on the institutional clinical guidelines.

### 2.5. Treatment plan selection using ProKnow scoring system and same-day CBCT

The proposed DT framework generates multiple candidate plans to facilitate optimal plan delivery on the treatment day. Each plan evaluation relies on the CBCT images acquired on the treatment day to ensure the accuracy of patients’ anatomy. To systematically assess plan quality, we adopted the ProKnow® (ProKnow Systems, Sanford, FL, USA) (Nelms *et al* 2012), which is a scoring method previously utilized in the 2016 AAMD/RSS-SBRT Prostate (Richard Sweat *et al* 2016) and plan quality studies (Gao *et al* 2023), and adapted the system for the two-fraction prostate proton SBRT treatment regimen. The ProKnow system assigns varying scores based on cumulative doses from CTV and OARs, with higher scores indicating superior clinical dosimetry outcomes compared to lower-scored plans. Using this system, we identify the optimal plan as the one achieving the highest scores among all candidate plans for the same patient at the same treatment fraction. Since the scoring system includes dose conformality metrics, we further define a volume (*V*) as an expansion from the CTV by 5 mm in all directions except posteriorly, using a 3 mm expansion. The *V* is used only for calculating dose conformality as the evaluation metrics in the ProKnow scoring system.

**Figure 3.**
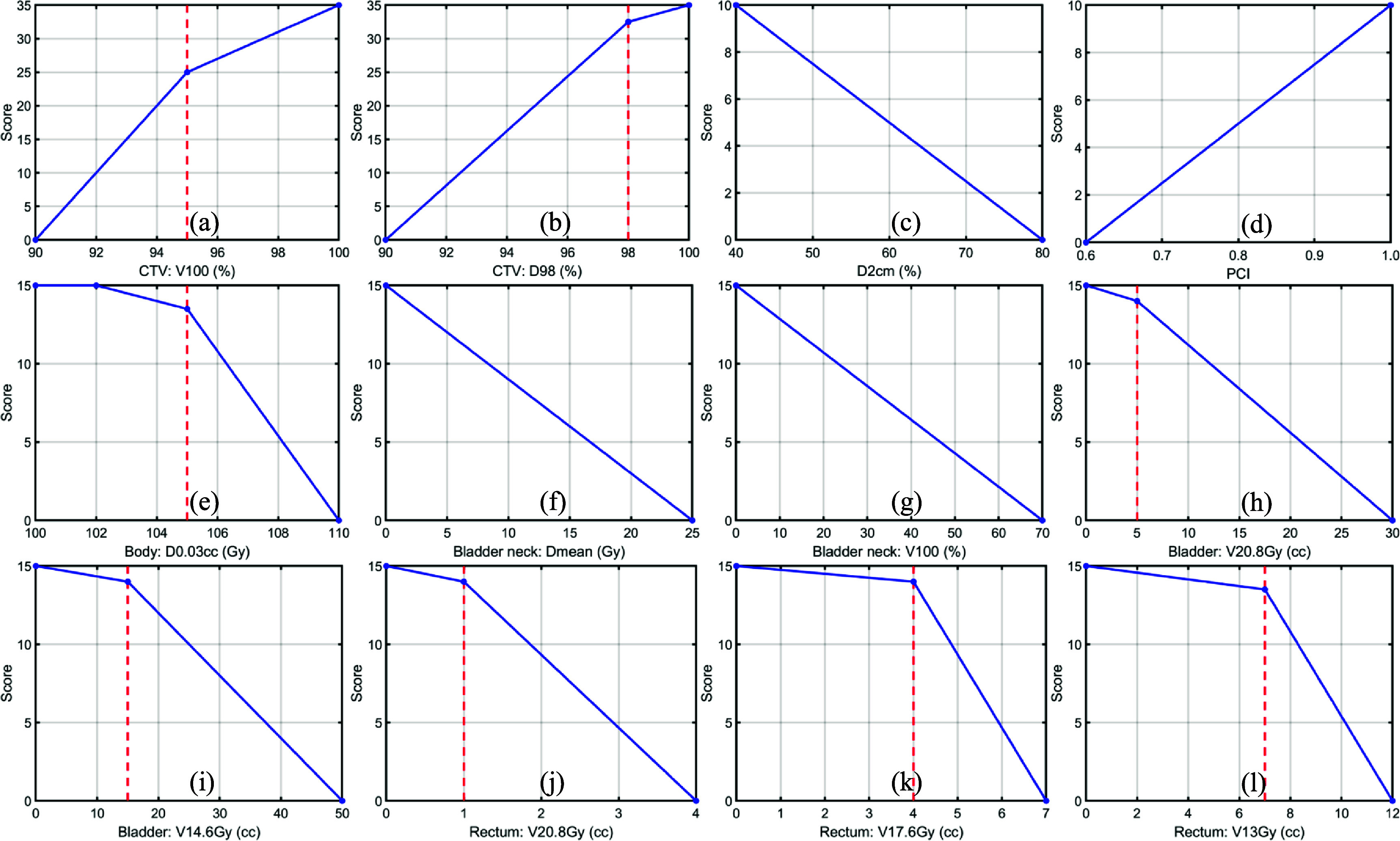
ProKnow scoring functions for quantifying the quality of clinical plans and DT-based plans. The scoring system is based on the cumulative doses from evaluated plans on the same-day CBCT, which shows the actual patients’ anatomy on the treatment day. (a)–(l) shows the scoring functions for different dosimetric parameters. The vertical red dashed lines depict the clinical planning goals.

Figure 3 illustrates our scoring system consisting of 12 scoring functions, adapted from the original ProKnow scoring functions based on our institutional planning guidelines and the reported two-fraction prostate SBRT clinical trials (i.e., 2STAR and 2SMART). Each scoring function corresponds to one plan quality metric. The involved plan quality metrics are V100 (the percentage of relative volume receiving 100% CTV prescription dose), D98 (the percentage of relative dose received by 98% CTV volume), D2cm (maximum dose at 2 cm from *V* relative to the prescription dose) which represent the dose fall-off speed, Paddick Conformality Index (PCI) (Paddick 2000), D0.03cc (dose received by the highest irradiated 0.03 cm^3^) which represents the hot spot of the proton plan, the mean dose and V100 of the bladder neck, V20.8 Gy and V14.6 Gy of the bladder, as well as V20.8 Gy, V17.6 Gy, and V13Gy of the rectum. We combined all scores from each metric to obtain the total score for plan quality evaluation. The vertical dashed lines in the figure denote the planning goals from table 1. Dose statistics for all Fx1 and Fx2 plans, as evaluated on the first and second treatment CBCT images, are detailed in tables A1–A3 (Appendix). The scores for each category in figure 3 can be derived from these tables. For example, table A1 indicates that the CTV V100 for Patient 1 under Clinic(Fx1) is 98.07%, and using figure 3(a), this corresponds to a score of 31.14. By summing the scores for all dosimetric parameters, the total ProKnow score for each plan can be calculated.

## 3. Results

### 3.1. Patient-specific CTV setup uncertainty

**Figure 4.**
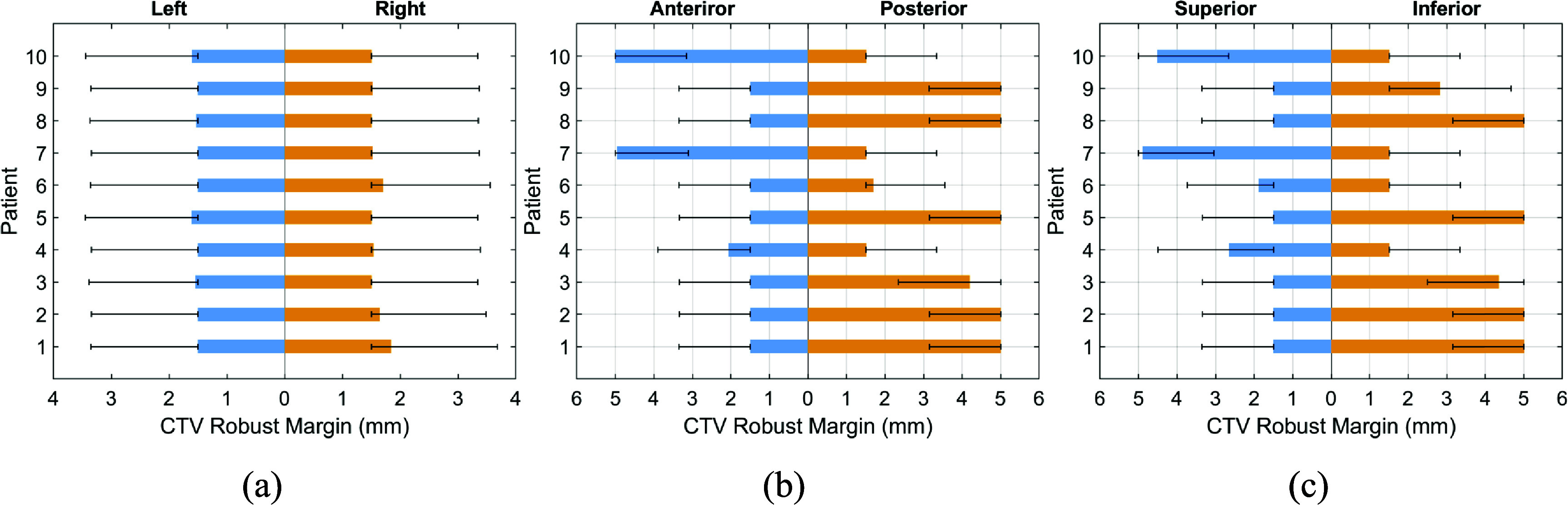
Patient-specific robust CTV setup uncertainty based on pCT for DT-based plans (treatment fraction 1) in (a) left-right, (b) anterior–posterior, and (c) superior-inferior directions. The error bars denote the uncertainty of ±0.5*σ* margins.

**Figure 5.**
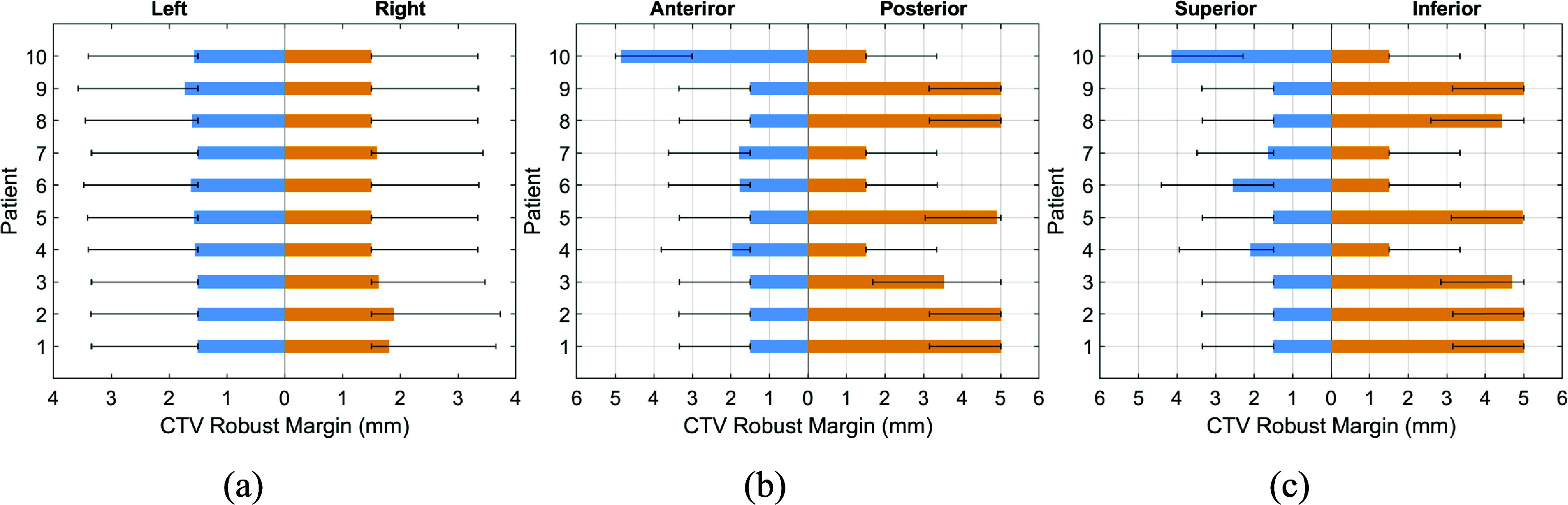
Patient-specific robust CTV setup uncertainty based on CB1 for DT-based plans (treatment fraction 2) in (a) left-right, (b) anterior–posterior, and (c) superior-inferior directions. The error bars denote the uncertainty of ±0.5*σ* margins.

Figure 4 illustrates patient-specific robust CTV setup uncertainty in the left-right, anterior–posterior, and superior-inferior directions, which were predicted by the trained GPR model using the features extracted from pre-treatment planning CT (pCT) images as depicted in figure 2 (Element 2). These setup uncertainties were employed by DT-based planning for the first treatment fraction. Error bars represent a range of ±0.5*σ* CTV uncertainty margins, bounded by 1.5 mm due to inherent uncertainties arising from the coincidence of radiation and mechanical isocenters. An additional 5 mm upper bound is imposed by our institutional clinical guidelines. Figure 5 displays the robust CTV setup uncertainty for all DT-based planning on the first treatment CBCT image set (CB1) utilized for the second treatment fraction.

### 3.2. DT-based plan selection for prostate SBRT fraction 1

**Figure 6.**
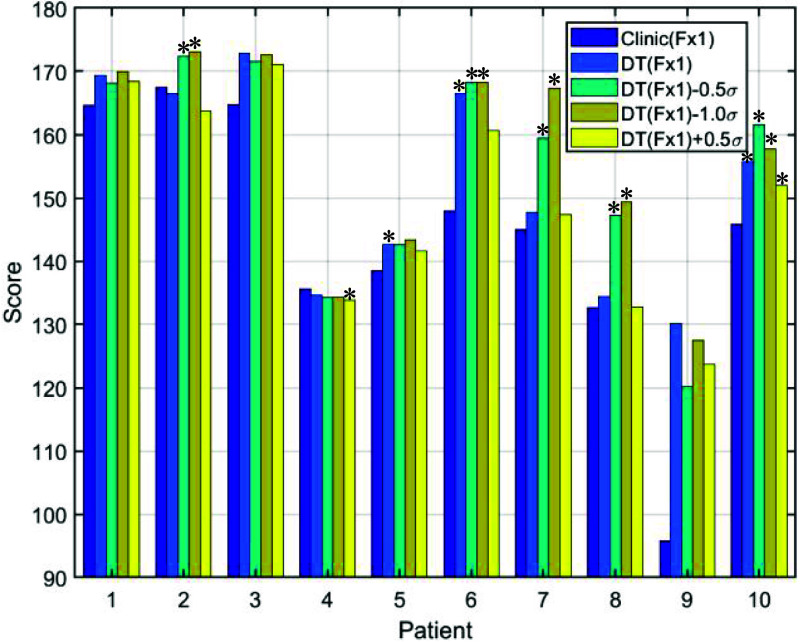
ProKnow score comparisons between the clinical and DT-based plans with different patient-specific CTV setup uncertainty. The plans were optimized on pCT, and the scores were derived based on the dosimetry parameters evaluated on CB1 from table A1 (Appendix) and scoring functions in figure 3. *The *p*-values (DT plans versus clinical plans) < 0.05.

For treatment Fx1, there are a total of 5 candidate plans including 1 clinical and 4 DT-based plans as depicted in figure 2 (Element 3). Table A1 (Appendix) presents the dose evaluation results of clinical and DT-based treatment plans for Fx1 calculated using CB1. The plans were generated utilizing proton robust optimization with pCT. Overall, both types of plans exhibit similar CTV coverage, with minor discrepancies. Notably, DT-based planning, particularly for Patient 9, demonstrates an 11% increase in V100 coverage compared to clinical planning. Additionally, the majority of DT-based plans showcase superior sparing of OARs compared to clinical plans. For instance, in Patient 3, the DT-based plan reduces bladder neck V100 by 18.2% compared to the clinical plan. Figure 6 illustrates the obtained plan scores calculated using the modified ProKnow scoring system, which demonstrates that most DT-based plans receive higher scores than clinical plans.

### 3.3. DT-based plan selection for prostate SBRT fraction 2

**Figure 7.**
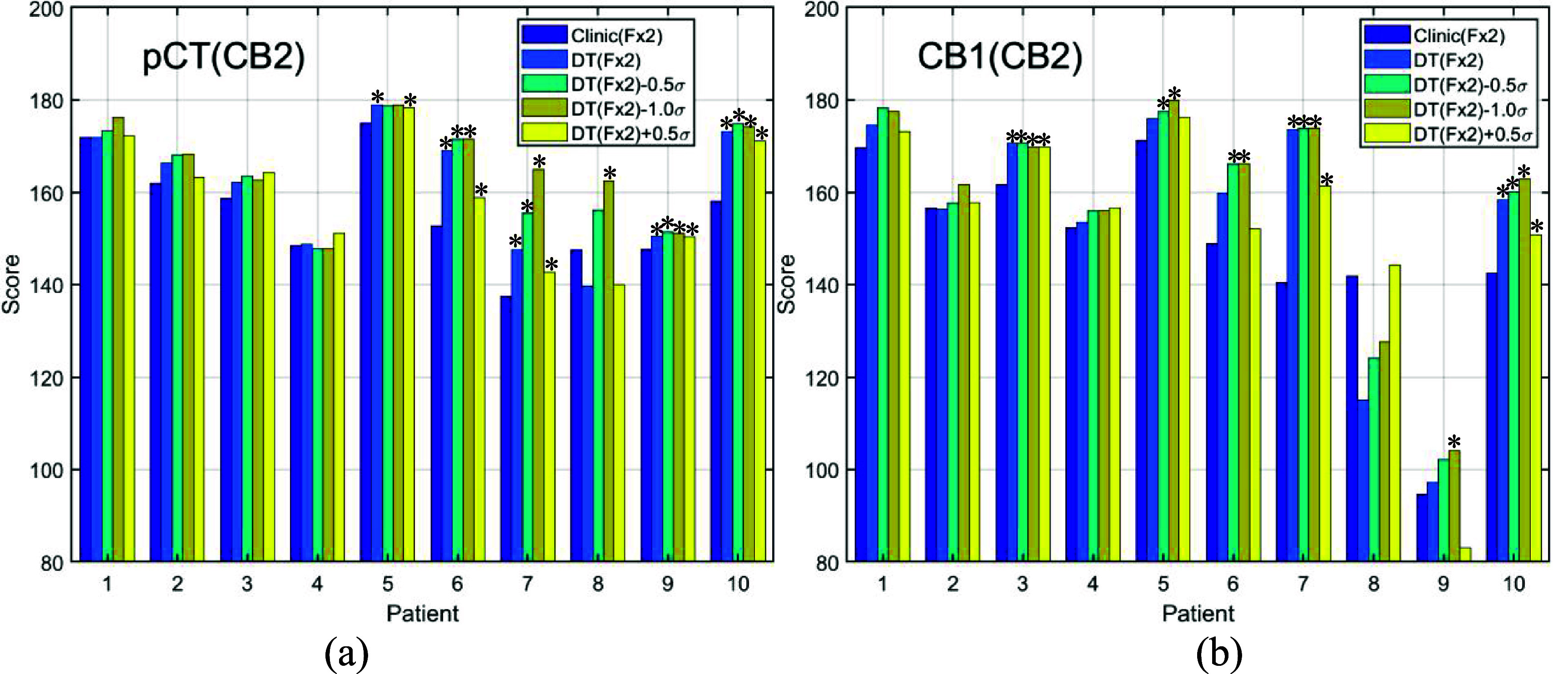
ProKnow score comparisons between the clinical and DT-based plans with different CTV setup uncertainty. (a) The plans were optimized on pCT, and the scores were derived based on the dosimetry parameters evaluated on CB2 from table A2 (appendix) and scoring functions in figure 3. (b) The plans were optimized on CB1, and the scores were derived based on the dosimetry parameters evaluated on CB2 from table A3 (appendix) and scoring functions in figure 3. *The *p*-values (DT plans versus clinical plans) < 0.05.

For Fx2 treatment, there are a total of 10 candidate plans, including 5 plans carried over from Fx1, as illustrated in figure 2 (Element 3). Table A2 compares the doses of the 5 plans carried over from Fx1 (i.e., the clinical and 4 DT-based treatment plans which were generated employing proton robust optimization with pCT) recalculated on the second treatment CBCT image set (CB2) for Fx2 evaluation. Similarly, table A3 assesses the actual doses of the other five treatment plans, which were optimized using CB1, recalculated on CB2. For each patient, the optimal treatment delivery plan is determined based on the obtained dosimetry endpoints listed in both tables. Figures 7(a) and (b) display the plan scores corresponding to each plan listed in tables A2 and A3, respectively. Notably, figure 7 reveals that 50% of the optimal plans originate from pCT-based plans, while the remaining 50% stem from updated planning images, CB1.

### 3.4. Comparisons of clinical and DT-based treatment plans using daily CBCT images

**Figure 8.**
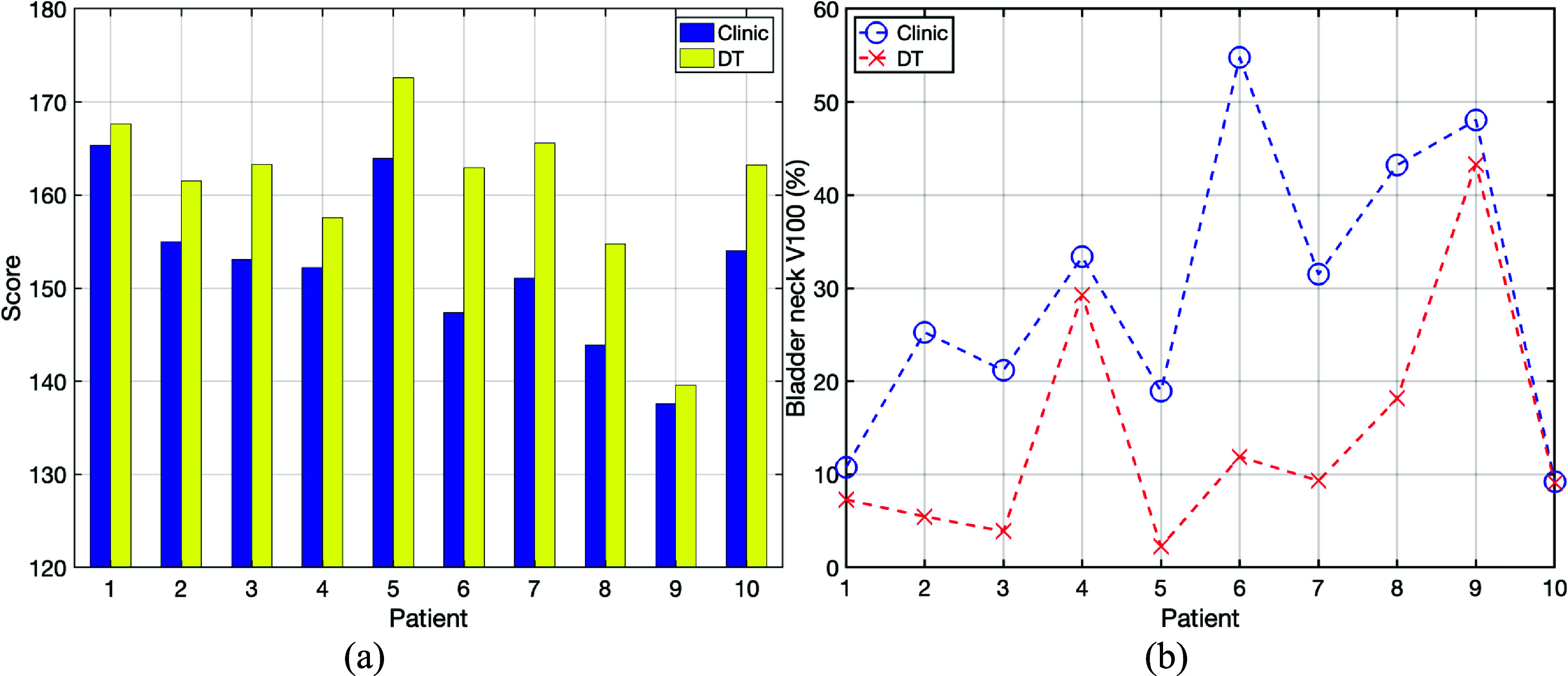
(a) ProKnow score comparisons between the clinical and DT-based plans based on the dose statistics derived from plan evaluation using CB1 for Fx1 and CB2 for Fx2. (b) Comparisons of V100 for the bladder necks between the clinical and DT-based plans. Detailed dosimetry parameters are given in table A4 (appendix).

Following the optimal of plans for Fx1 and Fx2, we assessed the overall treatment performance, as depicted in figure 2 (Element 7). Figure 8(a) shows the ProKnow score comparisons between the clinical and DT-based plans using the dosimetry parameters from plan evaluation for Fx1 and Fx2 together. Figure 8(b) depicts the comparisons of V100 for bladder necks between the clinical and DT-based plans. Table A4 provides a summary of dosimetry comparisons between clinical and DT-based plans for the complete two-fraction prostate SBRT treatment. Notably, for Patient 4, the DT plan enhances CTV V100 coverage by 0.54% and decreases body D0.03cc (hot spot), bladder neck V100 by 1.46%, and 4.08% compared to the clinical plan. For Patient 6, the DT-based plan decreases bladder neck V100 by 42.86% and bladder V14.6 Gy by 8.85 cc, compared to the clinical plan. Table A4 also indicates that the scores of the cumulative dose are higher for DT-based treatment regimen.

**Figure 9.**
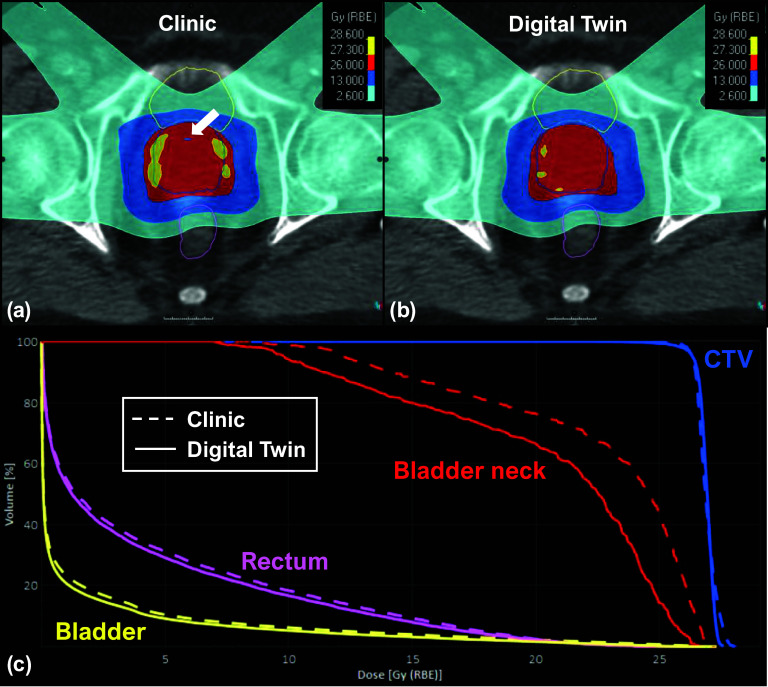
Dosimetry comparisons for Patient 3 between the clinical and DT-based plans. The top row displays the dose distributions in color wash for the (a) clinical plan and (b) DT-based plan, overlaid with transversal CT images and the contours of CTV (blue lines), bladder (yellow lines), and rectum (magenta lines). The bottom row (c) displays the dose-volume histogram (DVH) for CTV and OAR structures, where the dashed and solid lines represent the clinical plan and DT-based plan. The white arrow indicates the cold spot location in CTV.

**Figure 10.**
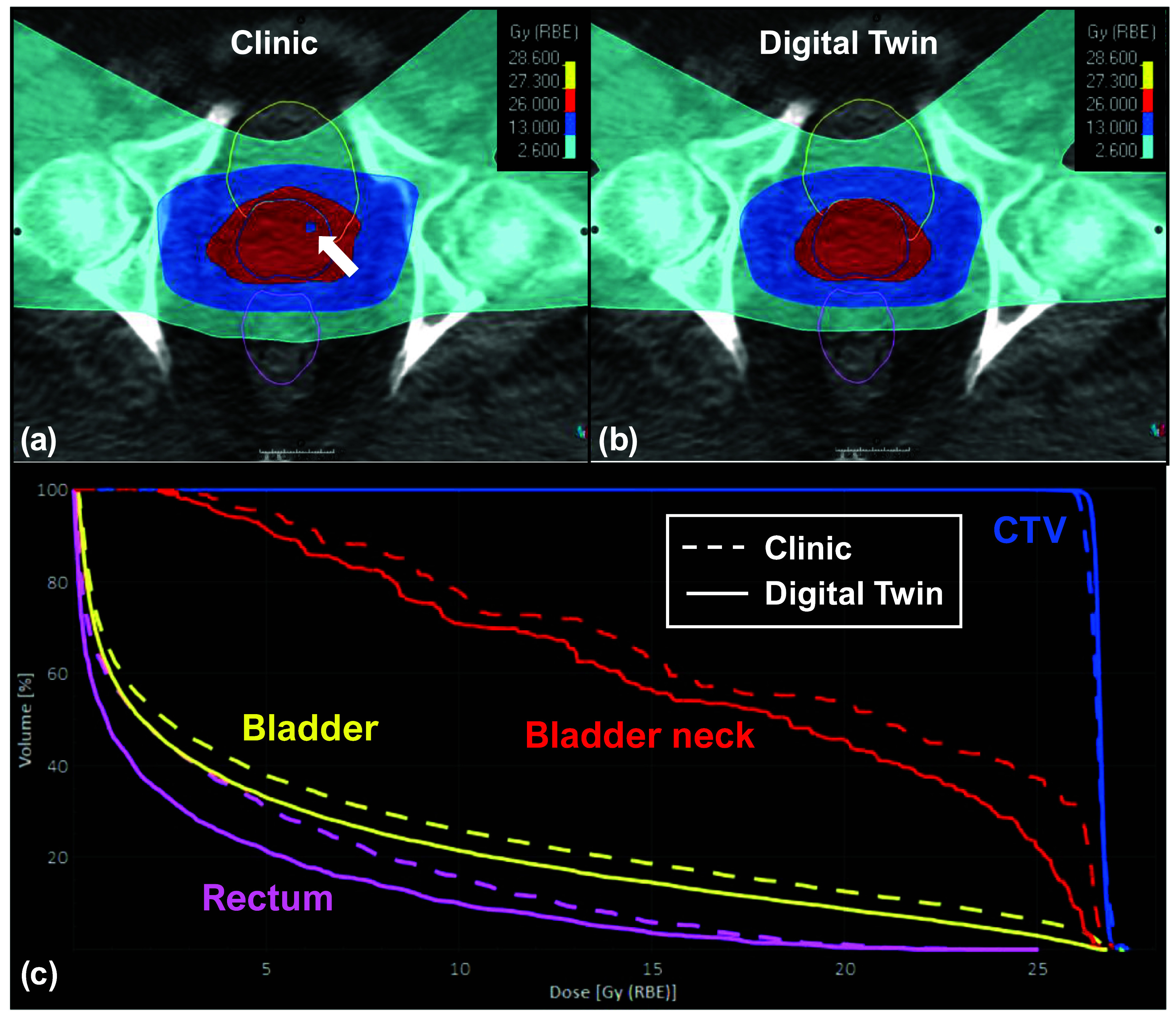
Dosimetry comparisons for Patient 7 between the clinical and DT-based plans. The top row displays the dose distributions in color wash for the (a) clinical plan and (b) DT-based plan, overlaid with transversal CT images and the contours of CTV (blue lines), bladder (yellow lines), and rectum (magenta lines). The bottom row (c) displays the dose-volume histogram (DVH) for CTV and OAR structures, where the dashed and solid lines represent the clinical plan and DT-based plan. The white arrow indicates the cold spot location in CTV.

**Figure 11.**
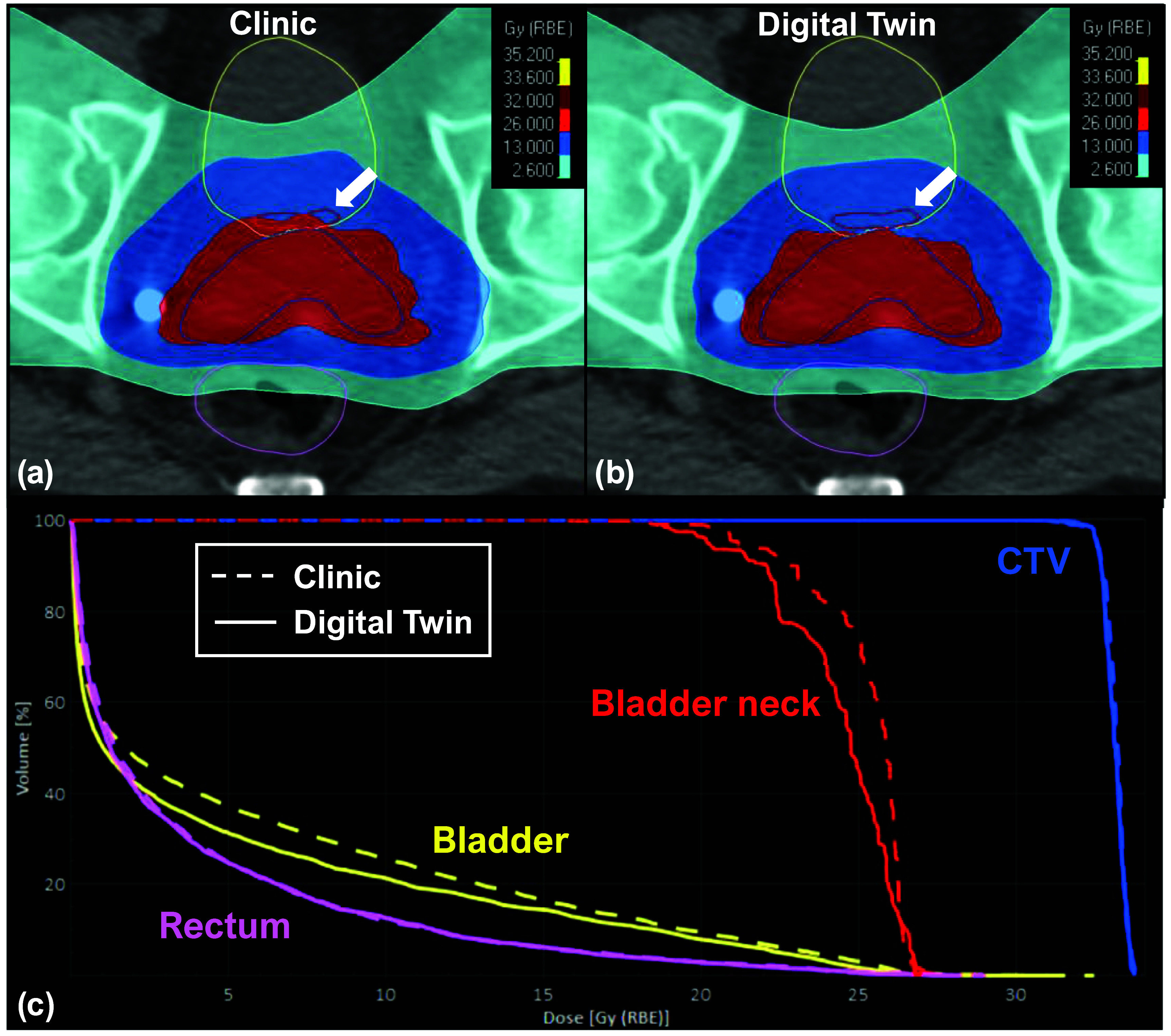
Dosimetry comparisons for Patient 8 between the clinical and DT-based plans. The top row displays the dose distributions in color wash for the (a) clinical plan and (b) DT-based plan, overlaid with transversal CT images and the contours of CTV (blue lines), bladder (yellow lines), bladder neck (red lines) and rectum (magenta lines). The bottom row (c) displays the dose-volume histogram (DVH) for CTV and OAR structures, where the dashed and solid lines represent the clinical plan and DT-based plan. The white arrow indicates that the clinical plan results in a significant portion of the bladder neck being covered by the full prescription dose.

Figures 9–11 depict the dosimetry comparisons between the clinical and DT-based plans. The dose calculations were performed using the cCBCT images acquired from Fx1 and Fx2 to consider the actual treatment setups. The clinical CTV setup uncertainties were 5 mm for all directions except for a posterior margin of 3 mm. The DT-based plan included various CTV setup uncertainties for each patient depending on the optimal plans selected for each treatment fraction. Figure 9 shows the patients with a 1.5 mm margin of CTV uncertainties in the left, right, anterior, and superior directions for Fx1 and Fx2. The posterior and inferior margins were 4.2 mm and 4.3 mm for Fx1, and the margins were 1.7 mm and 2.8 mm for Fx2, respectively. For patients in figures 10 and 11, the CTV setup uncertainties were 1.5 mm in all directions for Fx1 and Fx2. Figure 9(a) depicts dose inhomogeneity in the CTV with hot and cold spots for Patient 3 in the clinical plan. Furthermore, figure 9(c) demonstrates a significant reduction in doses to the bladder neck with the DT-based plan. Similarly, figure 10(a) displays a cold spot in the CTV and a high-dose region spilling into the bladder in the clinical plan for Patient 7, resulting in higher DVH values in figure 10(c), whereas the DT-based plan achieves lower doses to the rectum and bladder neck. Lastly, figure 11(a) shows greater anterior coverage in the clinical plan, leading to high-dose coverage for the bladder neck, consistent with DVH analyses in figure 11(c) for Patient 8.

## 4. Discussion

The proposed DT framework integrates pre-treatment CT, daily CBCT, patient-specific CTV setup uncertainty, and GPU-based MC TPS to enable the optimal treatment plan selection among the clinic and multiple DT plans. This selection is based on an online plan evaluation using the actual patient anatomy from CBCT at the time of treatment. The patient investigation results demonstrate that the DT plans can significantly reduce bladder neck doses in all 10 patients (table A4) while preserving CTV coverages, compared to the clinical plan, and without the need for re-planning due to bladder toxicity (Hathout *et al* 2014). The current clinical workflow requires re-planning if anatomical changes lead to insufficient CTV coverage or OAR overdoses. The institutional re-planning workflow takes 5 d for both conventional 28-fraction treatment and SBRT treatment. In general, the patient will be treated using the old plan until the new plan is ready. It is because treatment interruption can potentially impact local control and survival rates for patients (González Ferreira *et al* 2015). However, the treatment will be paused until the new plan is available for some special cases including SBRT, conventional treatment with a few fractions left, or physician requesting. In contrast, the proposed DT framework pre-generates multiple treatment plans based on the likelihood of potential patient-specific CTV setup uncertainty, supporting on-the-fly treatment decision-making. Compared to current clinical plans, the DT-based plans use smaller patient-specific robust CTV setup uncertainty, resulting in less dose to the surrounding healthy tissue. Figure 2 also shows that the proposed framework does not require online plan optimization, which can take up to 45 min using a conventional online adaptive proton therapy workflow with full multicriteria optimization for the presented case in this work. The proposed DT framework only requires the time for plan selection during the treatment (Element 4 in figure 2), which can be achieved in 2 min. By addressing the challenges of geometrical uncertainties and optimizing dose conformity, this approach has the potential to improve treatment outcomes, reduce normal tissue toxicity (Prasanna *et al* 2021), and ultimately enhance the quality of care for prostate cancer patients undergoing ultra-hypofractionated radiotherapy regimens.

Plan-of-the-day methods have been adapted to online adaptive proton therapy (Troost *et al* 2022), and this technique allows dose evaluation based on daily imaging to determine if dose constraints meet the requirement. This approach creates candidate treatment plans based on QACT, enabling plan selection for the remaining treatment fractions. On the other hand, the proposed DT framework includes two features: (1) planning on the existing CT and CBCT from each fraction and (2) using machine learning to make statistical inferences of the likely patient-specific CTV setup uncertainty. Planning on CBCT prevents patients from receiving extra CT exposure and waiting for CT scheduling. Combining (1) CBCT planning and (2) patient-specific setup uncertainty further augments the number of candidate plans for various treatment situations.

Figures 6 and 7 illustrate that the majority of DT-based plans achieve higher ProKnow scores compared to clinical plans, suggesting that the current clinical setup uncertainty for CTV might be overly conservative to demonstrate the advantages of proton therapy. The ProKnow scores are derived from the plan dose statistics presented in table A1–A3 (appendix) and the 12 scoring functions illustrated in figure 3. When all five plans have similar ProKnow scores, it reflects that the dose statistics across these plans are also comparable. For example, Patient 4 exhibits similar ProKnow scores across all five plans. Referring to table A1, it is evident that the dosimetric parameters for all 5 plans of Patient 4 are closely aligned, resulting in comparable ProKnow scores. For Patient 9 in figure 6, the ProKnow score for Clinic(Fx1) is significantly lower than others due to the undercoverage of CTV V100. As shown in table A1, the CTV V100 for Clinic(Fx1) is 87.59%, which is below the lowest threshold in figure 3(a), resulting in a score of 0 for CTV V100. In contrast, table A1 indicates that the CTV V100 for Patient 9 under DT(Fx1) is 98.59%, leading to a ProKnow score of 32.18, and DT(Fx1) exhibits a significantly higher score than Clinic(Fx1) in figure 6. Table A3 reveals that the CTV V100 values for Patient 9 are all below 90%, resulting in a score of 0 based on figure 3(a). Consequently, figure 7(b) illustrates that the scores for Patient 9 are relatively low, compared to other patients.

Figures 9–11(a) and (b) provide further evidence, showing that the areas covered by prescription doses and the 50% dose color wash are larger for clinical plans due to their wider CTV setup uncertainty compared to DT-based plans. These extended margins of setup uncertainty also pose challenges for the optimizer in finding a global solution during dose optimization. Figure 9(a) reveals dose heterogeneity in the CTV with hot and cold spots. In figure 10(a), a significant cold spot within the CTV is observed in the clinical plan, potentially increasing the risk of local failures. The DVH comparisons in figure 10(c) indicate higher doses to the bladder, bladder neck, and rectum compared to the DT-based plan. Figure 11(a) demonstrates that the clinical plan exhibits greater anterior prescription dose coverage, resulting in higher doses to the bladder and bladder neck, as confirmed by the dose color wash and DVH comparisons in figure 11(c).

**Figure 12.**
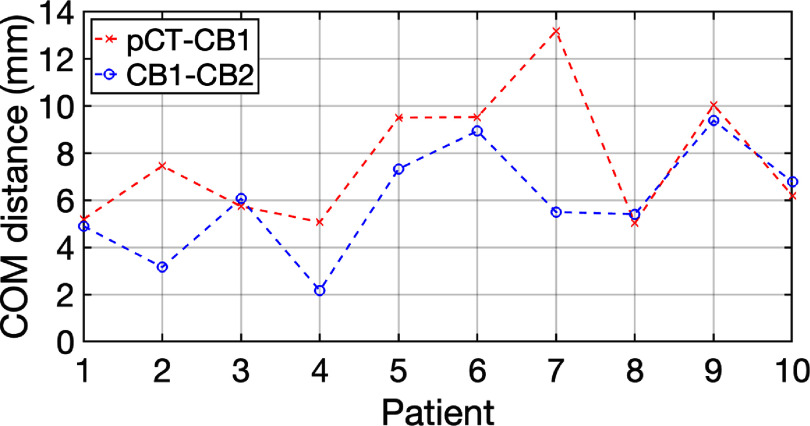
Comparisons of center of mass (COM) distances between pCT and CB1 (red) and between CB1 and CB2 (blue).

Figure 12 depicts the COM Euclidean distances of CTV contours between planning CT and each CBCT, and these distances can be used to surrogate patients’ anatomy changes. Although the COM distances range from 2.1 mm to 13.2 mm, table A4 indicates that DT-based plans yield lower doses of OAR, especially for bladder neck, and comparable or higher CTV coverages than clinical plans. DT-based plans achieved the lowest bladder neck V100 and Dmean for all patients as shown in figure 8(b), and bladder neck had been demonstrated to significantly impact the toxicity (Hathout *et al* 2014). According to the ProKnow scoring system, all DT-based plans outperform clinical plans regarding plan quality. As we focus on proton prostate SBRT, one of the current limitations is the numbers of investigated patients. Future investigation should include more prostate SBRT patients to fully demonstrate the advantage of DT-based treatment planning regarding target coverages and OAR sparing, especially for the bladder neck.

The proposed DT framework aims at achieving highly precise personalized RT. Two practical inquiries arise to assess the feasibility of this objective: (1) What quantity of candidate treatment plans should be considered? (2) How can the uncertainty in anatomy be effectively measured? Addressing the first query, we advocate for the inclusion of candidate plans from prior treatments in the plan database. Figure 2 illustrates five initial plans, derived from pre-treatment CT (pCT), as potential candidates for treatment administration. The number of candidate plans for Fx2 treatment increases to ten, comprising five plans based on CBCT and five from the initial pCT plans. Figure 7 demonstrates that for Fx2 treatment, 50% of the optimal plans originate from initial pCT plans, while the remaining 50% are based on CBCT plans. Figures 6 and 7(a) display varying optimal plans for patient 9 when assessing the same pCT plans using two distinct CBCT images, CB1 and CB2. For evaluation with CB1, the DT(Fx1) plan emerges as optimal, whereas DT(Fx2)-0.5*σ* is optimal based on CB2 evaluation. This outcome underscores the importance of incorporating previous candidate treatment plans into the database for future treatment deliberations.

Regarding the issue of anatomy uncertainty, the existing DT framework prioritizes ensuring that the CTV receives prescribed doses while minimizing doses to healthy tissues. To achieve this, GPR serves as a surrogate, consolidating prior knowledge of patient-specific CTV setup uncertainty within potential ranges. As depicted in table A4, DT-based plans demonstrate the ability to employ smaller CTV setup uncertainty, achieving comparable coverages to clinical plans while delivering reduced doses to OARs and minimizing hot spots. However, the effectiveness of the proposed framework is contingent upon the availability of CBCT images, which represent the patient’s actual anatomy on the treatment day, crucial for optimal plan determination. Additionally, the current framework incorporates a minimum 1.5 mm setup uncertainty from the coincidence of radiation and mechanical isocenter centers, this inherent uncertainty that could potentially be reduced with the implementation of more advanced quality assurance techniques for enhanced machine precision. The challenge of proton range uncertainty (Paganetti 2012), at 3.5%, is another significant consideration stemming from the limitations of CT material characterization methods, potentially compromising the conformity of proton therapy. Future research is likely to explore advanced deep learning-based material density mapping methods (Chang *et al* 2022a, 2022b, 2023a) to mitigate this uncertainty and minimize irradiation to normal tissues. A key focus of our future direction is to integrate multi-modality methods including advanced imaging techniques (Hussain *et al* 2022, Varoquaux and Cheplygina 2022) and large language models (Bhayana, 2024) into the framework to ensure the level of complexity and data accessibility can enhance the predictive capability of the proposed DT framework.

## 5. Conclusions

We demonstrated a framework harnessing the concept of DTs to elevate adaptive proton therapy for prostate SBRT. This framework optimized DT-based treatment plans utilizing the most probable patient-specific CTV setup uncertainty, which are smaller than our institutional standard values. Evaluation of plans based on corrected CBCT indicated that DT-based plans achieve superior or comparable CTV coverages while significantly sparing doses to the bladder neck, potentially reducing toxicity risks. By adapting the concept of DT, the proposed adaptive proton treatment framework has the potential to assist in treatment decision-making by identifying optimal solutions delivered for radiation oncology patients.

## Data Availability

The data cannot be made publicly available upon publication because they contain sensitive personal information. The data that support the findings of this study are available upon reasonable request from the authors.

## Appendix Dose volume endpoint comparisons of CTV and OAR for the ten cancer patients receiving two-fraction prostate SBRT.

Appendix includes dose volume endpoint comparisons of CTV and OAR from each treatment fraction of two-fraction prostate SBRT. Table A1 presents the dose evaluation results of clinical and DT-based treatment plans for Fx1 calculated using CB1. Table A2 compares the doses of the 5 plans carried over from Fx1 (i.e., the clinical and 4 DT-based treatment plans which were generated employing proton robust optimization with pCT) recalculated on CB2 for Fx2 evaluation. Similarly, table A3 assesses the actual doses of the other five treatment plans, which were optimized using CB1, recalculated on CB2. Table A4 provides a summary of dosimetry comparisons between clinical and DT-based plans for the complete two-fraction prostate SBRT treatment.

**Table A1.** Dose volume endpoint comparisons of CTV and OAR for the ten prostate SBRT patients, obtained by the clinical plans (clinic) and the plans by the proposed digital twin (DT) framework based on pCT images. Dose statistics were derived from plan evaluation using CB1 images before delivering treatment fraction 1 (Fx1). The σ denotes the uncertainty of CTV robust optimization margins for DT-based treatment planning.

pCT (planning) CB1 (Evaluation).		CTV.		Conformality .		Bladder neck.		Body.	Bladder.		Rectum.		
		V100 (%)	D98 (%)	D2cm (%)	PCI	V100 (%)	Dmean (Gy)	D0.03cc (%)	V20.8Gy (cc)	V14.6Gy (cc)	V20.8Gy (cc)	V17.6Gy (cc)	V13Gy (cc)
P01	Clinic(Fx1)	98.07	100.03	79.28	0.75	14.49	18.36	107.11	4.80	10.60	1.05	1.81	3.80
	DT(Fx1)	97.73	99.73	72.30	0.75	10.58	18.02	106.03	3.12	8.52	1.14	1.93	3.77
	DT(Fx1)-0.5*σ*	97.90	99.69	73.58	0.76	12.36	18.03	106.37	3.13	8.58	1.15	1.97	3.82
	DT(Fx1)-1.0*σ*	97.90	99.89	64.81	0.76	12.50	18.08	106.71	3.00	8.35	1.11	1.92	3.55
	DT(Fx1)+0.5*σ*	97.80	99.83	77.42	0.75	12.40	18.27	105.78	3.96	9.57	1.08	1.92	3.88

P02	Clinic(Fx1)	98.10	100.03	52.19	0.74	19.79	16.54	107.61	3.34	6.75	1.32	2.98	7.25
	DT(Fx1)	98.76	100.56	52.17	0.75	4.03	15.02	108.02	2.42	5.59	1.74	4.08	8.46
	DT(Fx1)-0.5*σ*	98.48	100.26	51.21	0.74	7.83	15.09	107.28	2.56	5.69	1.40	3.08	7.31
	DT(Fx1)-1.0*σ*	98.03	100.00	52.12	0.73	6.62	15.13	107.08	2.56	5.67	1.30	2.93	6.87
	DT(Fx1)+0.5*σ*	99.53	100.97	52.08	0.77	13.90	16.68	108.24	3.56	7.28	1.78	4.15	8.61

P03	Clinic(Fx1)	98.83	100.37	50.08	0.82	25.84	21.99	108.45	8.00	15.52	0.68	2.29	5.30
	DT(Fx1)	98.11	100.09	46.76	0.78	7.64	19.38	107.68	5.11	11.87	0.68	2.14	5.04
	DT(Fx1)-0.5*σ*	98.12	100.05	47.25	0.78	5.72	19.25	108.30	5.12	11.82	0.69	2.22	5.11
	DT(Fx1)-1.0*σ*	98.33	100.23	46.77	0.79	8.15	19.31	107.99	5.11	11.84	0.69	2.17	5.10
	DT(Fx1)+0.5*σ*	98.37	100.25	46.03	0.81	10.99	20.36	108.07	6.24	13.12	0.71	2.25	5.19

P04	Clinic(Fx1)	96.78	99.86	50.52	0.76	22.15	13.57	108.44	13.75	24.73	3.20	5.31	8.77
	DT(Fx1)	96.18	99.79	49.67	0.76	21.55	13.38	108.80	13.67	24.56	3.15	5.29	8.61
	DT(Fx1)-0.5*σ*	95.32	99.67	49.38	0.75	19.15	13.36	108.67	13.76	24.48	3.10	5.22	8.50
	DT(Fx1)-1.0*σ*	95.32	99.67	49.38	0.75	19.15	13.36	108.67	13.76	24.48	3.10	5.22	8.50
	DT(Fx1)+0.5*σ*	96.62	99.77	51.97	0.76	22.54	13.66	108.48	13.84	24.67	3.22	5.39	8.89

P05	Clinic(Fx1)	98.67	100.28	53.22	0.80	18.85	18.15	106.44	5.01	11.21	3.86	6.35	10.93
	DT(Fx1)	98.82	100.17	51.23	0.79	11.29	17.34	105.91	4.13	10.09	3.86	6.42	10.88
	DT(Fx1)-0.5*σ*	98.62	100.17	49.12	0.79	11.32	17.39	105.72	4.13	10.18	4.00	6.41	10.89
	DT(Fx1)-1.0*σ*	98.35	100.17	48.45	0.79	11.29	17.35	105.68	4.11	10.14	3.98	6.45	10.51
	DT(Fx1)+0.5*σ*	98.81	100.22	51.56	0.80	13.36	17.52	106.17	4.28	10.39	3.88	6.40	10.88
P06	Clinic(Fx1)	99.91	100.93	64.42	0.82	64.03	25.16	107.80	15.49	26.53	0.22	0.87	2.79
	DT(Fx1)	99.21	100.48	63.23	0.80	24.94	22.75	106.56	8.96	18.39	0.21	0.57	2.21
	DT(Fx1)-0.5*σ*	98.70	100.23	58.90	0.79	19.96	22.41	106.50	8.63	17.72	0.19	0.52	2.09
	DT(Fx1)-1.0*σ*	98.70	100.23	58.90	0.79	19.96	22.41	106.50	8.63	17.72	0.19	0.52	2.09
	DT(Fx1)+0.5*σ*	99.30	100.59	64.38	0.81	24.39	24.39	107.23	12.08	22.04	0.21	0.61	2.50

P07	Clinic(Fx1)	96.21	99.76	73.55	0.76	49.50	21.88	106.70	15.79	23.88	0.16	0.96	4.39
	DT(Fx1)	96.09	99.72	72.26	0.75	48.41	21.84	105.99	15.26	23.46	0.08	0.80	3.76
	DT(Fx1)-0.5*σ*	98.55	100.13	67.46	0.77	43.16	20.95	105.67	12.75	21.02	0.18	0.88	3.63
	DT(Fx1)-1.0*σ*	99.81	100.62	62.00	0.79	36.32	20.45	105.54	11.55	19.60	0.09	0.72	3.35
	DT(Fx1)+0.5*σ*	97.17	99.76	73.37	0.77	50.80	21.74	106.65	15.41	23.92	0.25	0.91	4.12

P08	Clinic(Fx1)	99.16	101.16	74.92	0.77	54.95	25.13	108.38	16.39	31.75	2.18	3.62	6.28
	DT(Fx1)	99.24	101.67	73.25	0.78	18.27	24.19	107.45	14.55	28.93	3.06	4.80	8.46
	DT(Fx1)-0.5*σ*	99.38	101.13	69.46	0.79	18.11	24.25	107.42	14.71	29.07	2.29	3.79	6.72
	DT(Fx1)-1.0*σ*	99.28	100.71	63.24	0.80	25.87	24.30	107.22	14.67	27.56	2.09	3.70	6.31
	DT(Fx1)+0.5*σ*	99.56	100.99	76.73	0.77	33.76	24.61	107.65	15.53	31.24	2.81	4.35	8.04

P09	Clinic(Fx1)	87.59	99.05	57.67	0.69	62.57	24.57	104.95	28.45	43.57	2.43	3.17	5.70
	DT(Fx1)	98.59	100.12	55.82	0.71	61.50	24.88	105.33	26.35	41.98	2.60	3.52	5.81
	DT(Fx1)-0.5*σ*	94.33	99.67	56.44	0.71	60.59	24.43	105.34	26.94	42.57	2.37	3.08	5.66
	DT(Fx1)-1.0*σ*	95.86	99.56	54.48	0.70	63.96	24.57	104.79	27.82	42.36	1.92	2.65	4.96
	DT(Fx1)+0.5*σ*	96.04	99.84	57.03	0.71	61.20	24.78	105.52	25.94	42.47	2.69	3.62	6.03

P10	Clinic(Fx1)	98.94	100.37	57.68	0.82	29.47	14.67	108.02	24.72	39.77	1.55	2.84	5.29
	DT(Fx1)	99.09	100.41	54.02	0.83	23.06	13.80	107.49	22.59	36.83	1.04	2.09	4.38
	DT(Fx1)-0.5*σ*	99.26	100.58	45.83	0.82	22.33	13.55	106.89	20.58	35.21	1.03	2.09	4.34
	DT(Fx1)-1.0*σ*	99.22	100.52	46.94	0.83	22.74	13.50	108.34	20.35	34.68	1.07	2.11	4.37
	DT(Fx1)+0.5*σ*	99.04	100.55	55.60	0.84	27.02	14.26	107.71	23.83	38.27	1.15	2.20	4.48

**Table A2.** Dose volume endpoint comparisons of CTV and OAR for the ten prostate SBRT patients, obtained by the clinical plans (clinic) and the plans by the proposed digital twin (DT) framework based on pCT images. Dose statistics were derived from plan evaluation using CB2 images before delivering treatment fraction 2 (Fx2). The σ denotes the uncertainty of CTV robust optimization margins for DT-based treatment planning.

pCT (planning) CB2 (Evaluation)		CTV		Conformality		Bladder neck		Body	Bladder		Rectum		
		V100 (%)	D98 (%)	D2cm (%)	PCI	V100 (%)	Dmean (Gy)	D0.03cc (%)	V20.8Gy (cc)	V14.6Gy (cc)	V20.8Gy (cc)	V17.6Gy (cc)	V13Gy (cc)
P01	Clinic(Fx2)	98.30	100.42	71.84	0.75	11.42	17.23	105.63	4.39	10.17	1.04	2.70	5.62
	DT(Fx2)	97.73	99.79	66.21	0.75	9.76	16.81	105.92	2.98	7.82	1.08	2.55	5.63
	DT(Fx2)-0.5*σ*	97.85	99.60	64.22	0.76	8.60	16.82	105.88	3.00	7.92	1.04	2.56	5.20
	DT(Fx2)-1.0*σ*	97.90	99.90	60.60	0.77	8.60	16.91	105.36	3.11	7.98	1.04	2.49	4.98
	DT(Fx2)+0.5*σ*	98.16	100.11	64.24	0.77	11.93	17.17	106.36	3.76	8.93	1.02	2.63	5.64

P02	Clinic(Fx2)	98.27	100.22	53.40	0.77	24.98	22.27	107.50	4.29	9.92	1.88	3.16	5.39
	DT(Fx2)	97.95	99.94	52.35	0.77	6.36	20.99	106.80	2.96	8.21	2.29	3.63	5.87
	DT(Fx2)-0.5*σ*	98.07	100.17	51.85	0.77	8.47	21.01	106.71	3.05	8.27	2.00	3.27	5.46
	DT(Fx2)-1.0*σ*	98.06	100.12	52.55	0.76	7.64	21.06	106.68	3.10	8.37	1.92	3.18	5.43
	DT(Fx2)+0.5*σ*	99.00	101.00	50.53	0.79	20.19	22.51	107.55	4.71	10.67	2.26	3.72	5.98

P03	Clinic(Fx2)	98.00	100.00	50.06	0.76	21.86	22.55	109.68	7.81	15.87	0.92	2.28	5.23
	DT(Fx2)	96.43	98.86	47.09	0.69	6.18	20.09	108.97	4.89	12.01	0.85	2.22	4.96
	DT(Fx2)-0.5*σ*	96.72	98.87	47.73	0.70	4.73	19.93	108.85	4.81	11.98	0.84	2.21	4.97
	DT(Fx2)-1.0*σ*	96.95	98.92	47.13	0.70	5.68	20.05	109.30	4.84	12.04	0.86	2.23	5.04
	DT(Fx2)+0.5*σ*	97.48	99.62	46.22	0.73	10.53	21.10	108.91	6.05	13.29	0.82	2.10	5.01

P04	Clinic(Fx2)	94.36	99.58	53.11	0.80	35.64	15.80	108.91	13.48	23.34	0.05	0.22	1.10
	DT(Fx2)	94.15	99.51	51.28	0.80	34.66	15.62	108.69	13.22	23.22	0.03	0.19	1.05
	DT(Fx2)-0.5*σ*	93.70	99.56	51.11	0.80	34.28	15.62	108.33	13.17	23.15	0.04	0.18	1.03
	DT(Fx2)-1.0*σ*	93.70	99.56	51.11	0.80	34.28	15.62	108.33	13.17	23.15	0.04	0.18	1.03
	DT(Fx2)+0.5*σ*	94.77	99.60	53.04	0.81	36.03	15.90	108.67	13.63	23.45	0.04	0.19	1.12

P05	Clinic(Fx2)	99.07	100.33	50.82	0.80	27.81	19.27	105.84	5.64	11.98	0.77	1.81	3.73
	DT(Fx2)	99.25	100.35	49.39	0.79	18.47	18.38	105.62	5.03	10.99	0.83	1.77	3.83
	DT(Fx2)-0.5*σ*	98.98	100.26	48.38	0.79	16.34	18.43	105.73	5.04	11.07	0.86	1.85	3.86
	DT(Fx2)-1.0*σ*	98.71	100.35	47.89	0.79	17.67	18.43	105.35	4.99	11.14	0.88	1.92	3.90
	DT(Fx2)+0.5*σ*	99.24	100.33	49.71	0.80	21.51	18.60	105.55	5.27	11.38	0.84	1.77	3.78
P06	Clinic(Fx2)	99.94	100.92	64.39	0.82	50.97	24.38	107.53	13.73	24.55	0.63	1.69	4.27
	DT(Fx2)	98.82	100.31	57.47	0.80	16.93	21.78	106.87	7.59	16.87	0.57	1.55	3.75
	DT(Fx2)-0.5*σ*	98.91	100.38	51.94	0.79	16.15	21.38	106.87	7.17	15.96	0.52	1.53	3.61
	DT(Fx2)-1.0*σ*	98.91	100.38	51.94	0.79	16.15	21.38	106.87	7.17	15.96	0.52	1.53	3.61
	DT(Fx2)+0.5*σ*	99.33	100.56	57.52	0.83	43.84	23.53	107.60	10.46	20.68	0.58	1.63	3.88

P07	Clinic(Fx2)	96.50	99.73	76.91	0.79	34.38	17.88	106.72	16.21	25.70	1.68	4.32	9.15
	DT(Fx2)	97.12	99.73	75.61	0.78	30.32	17.91	105.72	16.16	25.02	1.35	3.95	8.46
	DT(Fx2)-0.5*σ*	98.24	100.03	71.89	0.79	16.74	16.74	105.85	13.44	22.43	1.35	4.23	8.60
	DT(Fx2)-1.0*σ*	99.65	100.66	69.01	0.80	12.82	16.01	106.09	11.71	20.66	1.31	3.71	7.89
	DT(Fx2)+0.5*σ*	97.42	99.92	76.59	0.80	30.81	17.86	106.40	15.94	25.31	1.60	4.34	8.93

P08	Clinic(Fx2)	98.74	100.25	73.98	0.76	31.66	24.93	107.66	8.57	19.31	2.28	3.64	6.90
	DT(Fx2)	98.92	100.42	73.48	0.73	30.93	24.24	105.66	7.17	17.01	3.08	4.97	9.54
	DT(Fx2)-0.5*σ*	99.22	100.65	70.89	0.75	27.05	24.24	105.37	7.23	16.97	2.19	4.12	7.70
	DT(Fx2)-1.0*σ*	99.48	100.83	63.56	0.77	22.24	24.17	105.89	7.23	16.01	2.02	3.59	6.53
	DT(Fx2)+0.5*σ*	99.48	101.25	73.49	0.75	30.10	24.57	107.23	7.65	18.23	2.83	4.70	9.04

P09	Clinic(Fx2)	100.00	101.75	58.63	0.77	55.08	23.70	104.51	25.04	39.36	0.70	1.05	3.03
	DT(Fx2)	100.00	101.81	56.17	0.79	53.93	23.86	105.01	23.25	37.79	0.51	1.54	2.93
	DT(Fx2)-0.5*σ*	100.00	101.66	56.50	0.78	50.71	23.32	104.41	23.26	37.73	0.47	1.30	2.77
	DT(Fx2)-1.0*σ*	99.90	101.53	53.16	0.78	53.34	23.33	104.94	23.89	38.20	0.39	0.71	2.53
	DT(Fx2)+0.5*σ*	100.00	101.81	57.19	0.78	53.10	23.73	104.36	23.36	37.64	0.51	1.53	3.00

P10	Clinic(Fx2)	99.24	100.66	52.38	0.84	9.01	10.16	109.43	14.24	26.22	2.11	3.96	7.59
	DT(Fx2)	99.33	100.80	49.75	0.83	8.36	9.16	107.42	12.32	23.37	1.22	2.83	6.48
	DT(Fx2)-0.5*σ*	99.37	100.93	46.40	0.82	8.61	9.01	107.46	11.08	21.75	1.27	2.83	6.29
	DT(Fx2)-1.0*σ*	99.21	100.58	46.52	0.82	7.77	8.95	107.68	10.97	21.50	1.29	2.84	6.32
	DT(Fx2)+0.5*σ*	99.28	100.73	51.72	0.85	8.72	9.55	107.45	13.09	25.15	1.31	2.93	6.63

**Table A3.** Dose volume endpoint comparisons of CTV and OAR for the ten prostate SBRT patients, obtained by the clinical plans (clinic) and the plans by the proposed digital twin (DT) framework based on CB1 images. Dose statistics were derived from plan evaluation using CB2 images before delivering treatment fraction 2 (Fx2). The σ denotes the uncertainty of CTV robust optimization margins for DT-based treatment planning.

CB1 (planning) CB2 (Evaluation)		CTV		Conformality		Bladder neck		Body	Bladder		Rectum		
		V100 (%)	D98 (%)	D2cm (%)	PCI	V100 (%)	Dmean (Gy)	D0.03cc (%)	V20.8Gy (cc)	V14.6Gy (cc)	V20.8Gy (cc)	V17.6Gy (cc)	V13Gy (cc)
P01	Clinic(Fx2)	98.39	100.53	68.94	0.75	12.56	16.39	106.88	4.04	9.48	1.03	2.72	5.78
	DT(Fx2)	97.74	99.49	61.14	0.74	3.84	15.28	105.85	2.68	7.57	1.16	2.88	6.05
	DT(Fx2)-0.5*σ*	97.71	99.68	56.72	0.75	5.71	15.28	105.27	2.70	7.73	0.89	2.41	5.27
	DT(Fx2)-1.0*σ*	97.72	99.64	54.13	0.75	5.34	15.16	105.99	2.82	7.70	0.77	2.02	4.75
	DT(Fx2)+0.5*σ*	98.35	100.26	68.29	0.76	11.29	15.96	106.08	3.56	8.74	0.85	2.72	5.83

P02	Clinic(Fx2)	98.82	101.05	49.33	0.80	51.75	25.31	106.82	8.21	15.53	1.92	3.09	4.96
	DT(Fx2)	96.68	98.75	52.34	0.75	34.27	24.59	106.32	6.15	12.62	1.80	2.98	5.02
	DT(Fx2)-0.5*σ*	96.90	98.84	48.74	0.76	29.48	24.44	106.93	6.23	12.79	1.74	2.82	4.55
	DT(Fx2)-1.0*σ*	97.73	99.78	48.96	0.77	29.32	24.43	106.73	6.34	13.11	1.69	2.63	4.38
	DT(Fx2)+0.5*σ*	96.43	98.57	49.44	0.74	30.80	24.53	105.88	6.31	13.49	1.83	3.00	4.98

P03	Clinic(Fx2)	97.65	99.78	54.10	0.75	21.76	22.94	107.57	8.11	16.44	0.88	2.09	4.60
	DT(Fx2)	97.23	99.20	48.40	0.75	5.41	20.61	106.83	5.58	13.21	0.91	1.95	4.12
	DT(Fx2)-0.5*σ*	97.45	99.11	48.20	0.75	5.66	20.66	106.94	5.57	13.55	0.88	1.97	4.17
	DT(Fx2)-1.0*σ*	97.34	99.17	48.00	0.75	6.52	20.63	107.20	5.59	13.35	0.88	1.86	4.01
	DT(Fx2)+0.5*σ*	97.77	99.85	47.24	0.76	9.09	21.52	107.23	6.83	15.09	0.87	2.01	4.24

P04	Clinic(Fx2)	94.80	99.64	54.59	0.82	28.88	14.80	108.92	14.22	23.85	0.02	0.11	0.55
	DT(Fx2)	94.76	99.60	50.98	0.82	31.94	14.80	108.50	14.28	23.72	0.02	0.11	0.51
	DT(Fx2)-0.5*σ*	96.17	99.73	48.13	0.82	34.17	14.89	108.92	14.54	24.22	0.01	0.04	0.30
	DT(Fx2)-1.0*σ*	96.17	99.73	48.13	0.82	34.17	14.89	108.92	14.54	24.22	0.01	0.04	0.30
	DT(Fx2)+0.5*σ*	95.38	99.62	47.69	0.82	28.10	15.07	107.67	16.31	27.89	0.00	0.00	0.12

P05	Clinic(Fx2)	97.88	99.97	50.99	0.80	27.56	19.17	106.08	6.30	12.95	0.94	2.17	4.24
	DT(Fx2)	96.85	99.80	47.25	0.79	14.53	18.40	105.78	5.66	12.03	0.12	0.51	1.70
	DT(Fx2)-0.5*σ*	97.87	99.93	47.80	0.80	15.66	18.33	105.99	5.60	12.20	0.04	0.36	1.57
	DT(Fx2)-1.0*σ*	98.24	100.10	46.81	0.79	7.21	17.90	106.38	5.23	11.72	0.00	0.10	0.94
	DT(Fx2)+0.5*σ*	96.73	99.76	49.75	0.79	9.41	18.03	105.97	5.30	12.02	0.01	0.21	1.12
P06	Clinic(Fx2)	99.54	100.72	70.64	0.82	59.61	24.33	106.97	14.00	25.11	0.99	2.32	5.17
	DT(Fx2)	99.37	100.72	63.82	0.80	32.94	22.62	108.07	8.74	18.01	0.86	1.88	4.33
	DT(Fx2)-0.5*σ*	98.92	100.37	60.35	0.79	22.65	21.02	107.60	7.17	15.80	0.62	1.63	3.80
	DT(Fx2)-1.0*σ*	98.92	100.37	60.35	0.79	22.65	21.02	107.60	7.17	15.80	0.62	1.63	3.80
	DT(Fx2)+0.5*σ*	99.61	100.68	70.40	0.82	54.80	24.09	106.96	12.02	22.23	1.12	2.60	5.15

P07	Clinic(Fx2)	99.96	100.83	73.04	0.79	35.42	18.58	105.61	17.35	27.02	2.45	5.10	8.82
	DT(Fx2)	99.64	100.53	67.58	0.80	13.94	16.27	105.19	10.62	19.40	0.62	2.06	4.91
	DT(Fx2)-0.5*σ*	99.73	101.10	65.46	0.80	13.57	15.78	105.21	10.96	19.57	0.93	2.26	5.37
	DT(Fx2)-1.0*σ*	99.73	101.10	65.46	0.80	13.57	15.78	105.21	10.96	19.57	0.93	2.26	5.37
	DT(Fx2)+0.5*σ*	99.07	100.32	70.36	0.80	21.37	17.41	105.23	12.98	21.69	1.67	4.05	7.37

P08	Clinic(Fx2)	93.87	98.52	75.71	0.74	39.36	23.94	107.32	5.63	12.49	1.01	1.97	4.15
	DT(Fx2)	84.57	94.50	76.61	0.72	26.32	22.20	105.82	4.00	10.45	1.07	2.13	4.54
	DT(Fx2)-0.5*σ*	83.09	96.16	72.18	0.75	22.60	21.80	106.29	3.83	9.62	0.94	1.89	4.02
	DT(Fx2)-1.0*σ*	81.33	95.96	64.59	0.75	26.32	21.94	105.11	3.82	9.72	0.89	1.87	3.95
	DT(Fx2)+0.5*σ*	93.61	97.13	80.80	0.73	26.33	22.34	105.62	4.14	10.03	0.91	2.01	4.17

P09	Clinic(Fx2)	67.38	93.88	62.15	0.73	21.52	18.14	106.59	15.01	27.49	2.37	4.34	8.27
	DT(Fx2)	69.47	93.25	65.23	0.71	16.75	17.55	106.08	10.72	22.06	2.64	4.39	8.08
	DT(Fx2)-0.5*σ*	67.69	93.08	64.82	0.72	13.03	17.45	106.08	11.64	24.33	2.08	3.60	7.57
	DT(Fx2)-1.0*σ*	72.64	93.17	61.78	0.74	18.65	16.41	105.81	12.04	22.86	2.14	3.91	7.51
	DT(Fx2)+0.5*σ*	63.13	92.09	70.69	0.69	12.92	17.31	107.25	10.71	23.67	2.85	4.89	8.62

P10	Clinic(Fx2)	98.32	100.10	54.13	0.83	8.84	9.06	109.65	12.50	24.72	2.56	5.20	10.13
	DT(Fx2)	98.79	100.40	51.35	0.80	5.08	7.80	108.11	10.34	19.91	2.34	4.61	9.27
	DT(Fx2)-0.5*σ*	98.57	100.29	49.09	0.76	3.68	7.04	108.05	7.91	16.76	2.46	4.71	9.25
	DT(Fx2)-1.0*σ*	98.68	100.43	48.10	0.74	3.42	6.70	107.87	7.69	15.34	2.39	4.62	9.02
	DT(Fx2)+0.5*σ*	99.16	100.63	55.50	0.83	8.59	9.05	108.21	12.85	25.02	2.43	4.82	9.80

**Table A4.** Dose volume endpoint comparisons of CTV and OAR for the ten prostate SBRT patients, obtained by the clinical plans (clinic) and the optimal plans by the proposed digital twin (DT) framework from all fractions. Dose statistics were derived from plan evaluation using CB1 for Fx1 and CB2 for Fx2.

		CTV		Bladder neck		Body	Bladder		Rectum			Score
		V100 (%)	D98 (%)	V100 (%)	Dmean (Gy)	D0.03cc (%)	V20.8Gy (cc)	V14.6Gy (cc)	V20.8Gy (cc)	V17.6Gy (cc)	V13Gy (cc)	
P01	Clinic	97.46	99.58	10.73	18.46	105.03	4.91	11.11	0.83	1.95	4.81	165.37
	DT	97.60	99.25	7.29	17.36	104.33	3.28	8.79	0.79	1.67	4.19	**167.64**

P02	Clinic	98.25	100.22	25.27	22.15	106.63	4.68	11.43	1.50	2.80	5.42	154.99
	DT	97.76	99.61	5.48	20.63	105.69	3.31	9.44	1.53	2.76	5.33	**161.53**

P03	Clinic	98.59	100.42	21.17	22.47	107.76	8.33	17.29	0.70	2.16	5.20	153.08
	DT	97.70	99.68	3.89	20.38	106.03	5.63	13.81	0.64	1.88	4.58	**163.30**

P04	Clinic	99.35	100.22	33.36	16.08	107.65	13.23	22.48	0.25	1.23	4.11	152.22
	DT	99.89	100.54	29.28	15.72	106.19	14.38	24.48	0.01	0.34	2.90	**157.60**

P05	Clinic	99.32	100.68	18.94	18.70	105.55	4.85	10.99	1.41	2.65	5.32	163.96
	DT	98.92	100.85	2.25	17.68	104.31	4.33	10.12	0.25	1.24	3.58	**172.59**

P06	Clinic	99.92	101.48	54.76	24.30	105.67	13.89	25.79	0.35	1.36	4.06	147.38
	DT	98.59	100.50	11.90	21.53	105.77	7.47	16.94	0.17	1.00	2.94	**162.91**

P07	Clinic	99.21	100.27	31.51	18.39	105.33	17.89	29.30	0.37	1.97	6.27	151.08
	DT	99.69	101.32	9.32	16.77	104.00	12.12	22.90	0.25	1.06	4.17	**165.56**

P08	Clinic	98.88	101.14	43.22	25.18	106.01	9.48	19.15	2.26	3.72	6.58	143.89
	DT	99.07	101.44	18.17	24.38	105.28	7.97	16.30	2.10	3.45	6.53	**154.77**

P09	Clinic	99.85	100.63	48.04	23.45	103.95	25.55	42.07	0.89	2.18	3.60	137.61
	DT	99.69	101.26	43.32	23.31	103.54	23.88	40.38	1.08	1.95	3.94	**139.62**

P10	Clinic	99.16	100.79	9.25	10.40	106.70	16.89	29.14	1.54	3.19	6.27	154.02
	DT	99.24	101.03	9.08	9.32	106.23	12.76	24.84	0.90	2.28	5.22	**163.26**
